# SPOUSAL INFLUENCE ON EARLY RETIREMENT DECISIONS: ORIGINS AND MECHANISMS

**DOI:** 10.1093/geroni/igz038.164

**Published:** 2019-11-08

**Authors:** Maria Eismann, Kène Henkens, Matthijs Kalmijn

**Affiliations:** 1 NIDI, The Hague, Netherlands; 2 Netherlands Interdisciplinary Demographic Institute, South-Holland, Netherlands; 3 University of Amsterdam, Amsterdam, Netherlands

## Abstract

The interdependence between partners raises considerable interest in the sociology of life course, work and families. Partner influences play a particularly important role in the work domain, because each partner’s work decisions have profound effects on the couple as a whole. In contrast to previous research, this study pays detailed attention to the role partners play in workers’ labor market decisions by using the case of early retirement decisions. We hypothesize that partners’ preferences for older workers’ retirement originate from altruism and self-interest. For example, partners might prefer workers to retire early because the worker’s job is highly stressful or partners might prefer workers to retire early to increase possibilities for joint leisure. Moreover, we expected that partners influence older workers’ early retirement behavior via persuasion and pressure. So, partners might either convince workers to change their preferences, or they might pressure workers to act according to the partners’ preferences, irrespective of workers’ own preferences. To adequately estimate partners’ and workers’ preferences for workers’ early retirement, we used an instrumental variable approach. This was possible due to the multi-actor longitudinal data available from a large representative sample of older workers and their partners in the Netherlands. The results support that partners’ preferences originate in altruism and self-interest and that partners influence workers through persuasion and pressure. Gender differences were marginal, with stronger signs for altruistic origins among female than male partners.

